# Clinico-pathological profile of lung cancer in Uttarakhand

**DOI:** 10.4103/0970-2113.53229

**Published:** 2009

**Authors:** Jagdish Rawat, Girish Sindhwani, Dushyant Gaur, Ruchi Dua, Sunil Saini

**Affiliations:** *Department of Pulmonary Medicine, Himalayan Institute of Medical Sciences, Dehradun, Uttarakhand, India*; 1 *Department of Pathology, Himalayan Institute of Medical Sciences, Dehradun, Uttarakhand, India*; 2 *Department of Oncology, Himalayan Institute of Medical Sciences, Dehradun, Uttarakhand, India*

**Keywords:** Lung cancer, histopathology type, smoking, squamous cell carcinoma

## Abstract

**Background:**

Lung cancer is one of the most aggressive and prevalent type of malignancy causing high morbidity and mortality. Tobacco smoking continues to be the leading cause of lung cancer worldwide. An increasing incidence of lung cancer has been observed in India.

**Objective:**

The aim of this study was to evaluate the clinico, a pathological profile of the lung cancer in hilly state of Uttrakhand.

**Materials and Methods:**

We performed a retrospective analysis of histopathologically proven cases of bronchogenic carcinoma admitted in our hospital from January 1998 to August 2005.

**Results:**

Our study included 203 patients with confirmed cases of lung cancer. Male to female ratio was 8.2:1. The common age group being 40-60 years, 9.86% of the patients were less than 40 years old age. Smoking was found to be the main risk factor in 81.77% patients. The most frequent symptom was cough (72.90%) followed by fever (58.12%). The most common radiological presentation was mess lesion (46.31%). The most common histopathological type was squamous cell carcinoma (SCC) (44.83%) followed by adenocarcinoma (19.78%) and small cell lung carcinoma (SCLC) (16.75%). The majority patients (73.29%) were diagnosed in the later stages of the disease (III B and IV).

**Conclusion:**

It was found out that SCC was the most frequent histopathological form. SCLC predominates below 40 year and SCC over 60 years of age. Smoking still remains the major risk factors in pathogenesis of lung cancer.

## INTRODUCTION

Lung cancer is believed to be the most common fatal neoplastic disease in the world today. It is responsible for 28% of all the cancer related deaths.[1] In the developed countries, incidence and mortality from lung cancer in females is rising, whereas it is declining in males. Lung cancer is responsible for approximately one million deaths per year at present, and it is estimated to rise to three million per year by the year 2010.

Progressive survival extension and increasing cigarette smoking has led to a numerical rise of patients with primary lung cancer in India. It is in accordance with the epidemiological data from western countries, which shows rising prevalence of the disease in Indian population.[2] Smoking is the cause for more than 85% of the bronchogenic carcinoma cases.[3,4] According to the world health organization (WHO) classification formulated in 1999; there are six major types of malignant epithelial non-small cell lung carcinoma (NSCLC) and small cell lung carcinoma (SCLC).[5] The proportions of histopathological cell types of lung cancer vary with changes in social and other environmental factor.

We undertook this retrospective review of patients diagnosed with lung cancer at Himalayan Institute of Medical Sciences (HIMS), the only postgraduate institute and a large tertiary acre center of the region, to understand the clinico in this hilly state.

## MATERIALS AND METHODS

This retrospective study was performed using a database with 203 patients of lung cancer who had been diagnosed at our hospital, during January-August 2005.

The clinical records of the patients were received for demographic data, smoking history, duration of symptoms, symptoms and signs, radiographic findings, histopathology, and clinical staging of lung cancer. Only patients with a confirmed pathological cell type and adequate medical records were included for the analysis. For confirmation of diagnosis of lung cancer, majority of patients were subjected to fiber-optic bronchoscopy and/or percutaneous fine needle aspiration biopsy (FNAB) under imaging guidance.

The Ethical committee of the Institute has approved the study.

## RESULTS

The series included 181 male (89.16%) and 22 female (10.84%) patients. Age distribution of these patients is shown in [Table 1]. In less than 40 years of age, SCLC was the commonest type, while SCC was common (71.69%) after 60 years of age. The break up of both sexes according to their smoking history is shown in Table 1. Cough was the most common symptom found in (72.90%) patients, followed by fever (58.12%), chest pain (55.64%), and dyspnea (50.74%) [Table 2].

**Table 1:** Demographic profile of cases included in the study (n = 203)

Age group	No. of patients	Percentage	
<40	20	9.86	
41–60	108	53.20	
>60	75	36.94	
Total	203	100	
Gender			
Male	181	89.16	
Female	22	10.84	
Total	203	100	
Smoking status	Male	Female	M:F
Smoker	161	5	32.2:1
Nonsmoker	20	17	1.2:1
Total	181	22	8.2:1

Mass lesion (46.13%) was the commonest radiological feature followed by collapse-consolidation (40.89) [Table 2]. The various diagnosis modalities, either single or in combination, used for confirmation of lung cancer are shown in [Table 3]. Central endobronchial tumors were seen in 99 (48.77%) patients, whereas peripheral tumors in 104 (51.23%). The adenocarcinoma most commonly manifested as peripheral mass (75%).

**Table 2:** Clinico- radiological manifestations (n = 203)

Symptoms	No. of patients	Percentage
Cough	148	72.90
Fever	118	58.12
Hemoptysis	51	25.12
Chest pain	113	55.67
Dysponea	103	50.74
Hoarseness of voice	38	18.72
SVC obstruction	24	11.82
Loss of weight and appetite	115	56.65

**Table:** Radiological presentation

Site	Number	%
Bilateral	6	2.96
Right lung	117	57.64
Left lung	80	39.41
Mass	94	46.31
Collapse-consolidation	83	40.89
Pleural effusion	9	4.43
Combined presentation	17	8.37

The most common histopathological type was SCC (44.83%), followed by adenocarcinoma (19.70%), and SLCC (16.75%) [Table 3]. The majority of patients (73.29%) were diagnosed in the later stages of the disease. The patients presented to their physician, on an average, 112 days (range 30-270), after the onset of symptoms.

**Table 3:** Diagnostic yield of various investigative procedures

Diagnostic procedure	NSCLC	SCLC	Diagnostic yield (%)
FOB	81	18	48.77
Lymph node biopsy	4	-	1.97
P/C TT FNAC	73	16	43.84
Pleural biopsy	9	-	4.43
Pleural fluid	2	-	0.99
Total	169	34	100

**Table:** Histological type of lung cancer

Histology	No. of patients	%
Squamous cell carcinoma	91	44.83
Adenocarcinoma	40	19.70
Large cell carcinoma	17	8.37
Undifferentiated carcinoma	21	10.34
Small cell carcinoma	34	16.75
**Total**	203	100

## DISCUSSION

Most of our study belonged to the patients of age group between 40-60 years, with a male predominance (M:F ratio 8.2:1). Smoking was the most common predisposing factor, which included cigarettes, beedis, hookah, etc. Similar observation has been reported by other Indian studies also.[6–10] In contrast to our study, Belcher JR[11] found that in USA and UK, the male: female ratio was approximately 5:1 in 1970 but fell to around 2.5:1 in 1982. This is because of the striking increase in cigarette smoking in western women.

The prevalence of respiratory system malignancies is quite variable in different parts of India. In most studies, including reports of National Cancer Registry Program (NCRP)[12,13] from Bhopal, Delhi, and Mumbai; and other studies;[12,14] laryngeal cancer was the most common site. The present study was in agreement with those where lung was the topmost site for malignancy in males.

One important observation made in our study is the delay in presentation of patients to their attending physician. Majority of the cases were misdiagnosed as tuberculosis and treated at various other centers, there by causing a delay in diagnosis.

In our study, the delay in seeking treatment was observed to vary from 4-6 months, which is similar to another study.[15] The diagnosis of cancer in an individual not only affects the person physically but produces significant psychological disturbance too. A study from Tata Memorial Hospital, Mumbai,[16] analyzing the psychological state of patients suffering from cancer, concluded that 89% of the patients used denial as a mental defense mechanism, leading to delay in seeking medical help for the confirmation and treatment of cancer. Psychological reasons such as, denial of illness, fear of cancer, fear of its treatment, and domestic difficulties were the most common causes of delay in seeking treatment. This emphasizes the need for patient counseling as well as more effective methods for early detection of lung cancer cases by general practitioner.

Our data shows that unexplained cough of several weeks is the commonest symptoms along with fever, weight loss, chest pain, and shortness of breath. This is similar to reports published in the literature from different part of India.[10,17,18]

The pattern of lung cancer has been changing in the West. Lung cancer is being increasingly diagnosed in women and adenocarcinoma has over taken SCC as the most common histological cell type.[19] However, the pattern seen at our hospital was different. SCC was still the commonest cell type seen, followed by adenocarcinoma and SLCC. This is similar to reports from other part of India.[6–9,17,18] This difference in histopathology may be due to the fact that smoking is less prevalent among women in India as opposed to the West, where it is rising; and urbanization, that exposes the patient to other carcinogens, risk factors or a complex interaction among gender, race, smoking status in West.

Bronchoscopy is the most useful investigation in the evaluation of the patient suspected of endobronchial lung cancer. Tumors that were beyond bronchoscopic vision, are difficult to reach and require the other technique.[20] FNAB done under CT is the investigation of choice for peripherally situated lesions, which has very high complication rates as seen in various international,[21–23] and Indian studies.[24,25] In our study, the overall yield with bronchoscopy was 48.77% and with FNAB was 43.84%.

The commonest radiological finding seen in present study was mass followed by collapse consolidation with slight predominance of right lung; similar to reports published in the literature.[10,26] The adenocarcinoma commonly manifested as peripheral mass or a malignant pleural effusion. Similar finding were also reported in other studies.[27,28] The SCLC presented commonly as central lesion, which was in agreement with other studies.[19,27,29]

## CONCLUSION

This study has shown smoking as the principle risk factor in the causation of lung cancer among men. Primary lung cancer should always be suspected in a person presenting with unexplained cough of several weeks with other symptoms such as weight loss and fever with nonresolving collapse-consolidation on chest radiograph; and further investigations should be carried out to rule lung cancer. Majority of the cases were misdiagnosed as tuberculosis and treated by antitubercular treatment, thereby causing delay in diagnosis, this emphasized the need for more effective methods for early detection of lung cancer cases among general population.
